# Identifying student opinion leaders to lead e-cigarette interventions: protocol for a randomized controlled pragmatic trial

**DOI:** 10.1186/s13063-020-04990-z

**Published:** 2021-01-06

**Authors:** Kar-Hai Chu, Sara Matheny, Alexa Furek, Jaime Sidani, Susan Radio, Elizabeth Miller, Thomas Valente, Linda Robertson

**Affiliations:** 1 University of Pittsburgh, Pittsburgh, PA USA; 2 UPMC Hillman Cancer Center, Pittsburgh, PA USA; 3 UPMC Children’s Hospital, Pittsburgh, PA USA; 4 University of Southern California, Los Angeles, CA USA

**Keywords:** Social network analysis, Tobacco, School, E-cigarette

## Abstract

**Background:**

After the US Surgeon General declared youth electronic cigarette (e-cigarette) use an epidemic in 2018, the number of youth e-cigarette users continued to surge, growing from 3.8 million in 2018 to over 5 million 2019. Youth who use e-cigarettes are at a substantially higher risk of transitioning to traditional cigarettes, becoming regular cigarette smokers, and increasing their risk of developing tobacco-related cancer.

A majority of youth are misinformed about e-cigarettes, often believing they are not harmful or contain no nicotine. Middle school students using e-cigarettes have been affected by its normalization leading to influence by their peers. However, social and group dynamics can be leveraged for a school-based peer-led intervention to identify and recruit student leaders to be anti-e-cigarette champions to prevent e-cigarette initiation. This study outlines a project to use social network analysis to identify student opinion-leaders in schools and train them to conduct anti-e-cigarette programming to their peers.

**Methods:**

In the 2019–2020 academic school year, 6th grade students from nine schools in the Pittsburgh area were recruited. A randomized controlled trial (RCT) was conducted with three arms—expert, elected peer-leader, and random peer-leader—for e-cigarette programming. Sixth grade students in each school completed a network survey that assessed the friendship networks in each class. Students also completed pre-intervention and post-intervention surveys about their intention-to-use, knowledge, and attitudes towards e-cigarettes. Within each peer-led arm, social network analysis was conducted to identify peer-nominated opinion leaders. An e-cigarette prevention program was administered by (1) an adult content-expert, (2) a peer-nominated opinion leader to assigned students, or (3) a peer-nominated opinion leader to random students.

**Discussion:**

This study is the first to evaluate the feasibility of leveraging social network analysis to identify 6th grade opinion leaders to lead a school-based e-cigarette intervention.

**Trial registration:**

ClinicalTrials.gov NCT04083469. Registered on September 10, 2019.

**Supplementary Information:**

The online version contains supplementary material available at 10.1186/s13063-020-04990-z.

## Background

Electronic cigarette (e-cigarette) use has been rapidly growing among adolescents. The 2019 National Youth Tobacco Survey found current e-cigarette use among middle school students increased from 4.9 to 10.5% [1]. This increase reversed years of decline in overall youth tobacco product use and occurred despite the US Surgeon General declaring e-cigarette use among youth a national epidemic the year prior [2]. Studies have consistently found that e-cigarettes may facilitate the uptake of traditional cigarette smoking among otherwise never-smoking youth [3–7]; additionally, a recent outbreak of severe pulmonary illness has been linked to e-cigarette use.

Nationally representative data regarding perceptions, knowledge, and attitudes surrounding e-cigarettes suggest a simultaneous need and opportunity to conduct school-based education programs on e-cigarettes for youth. Approximately 80% of youth do not think regular e-cigarette use is harmful [8], and many have limited awareness of nicotine content. The e-cigarette company JUUL, which owns 70% of the e-cigarette market, advertises enhanced nicotine delivery; yet in a sample of 15–24 year olds who used a JUUL product in the past 30 days, only 37% knew that its cartridges always contain nicotine [9]. Awareness is also lacking for parents and teachers, increasing the risk of continued misinformation being provided to youth. Focus groups have found that most adults are generally unaware of the ingredients in e-cigarettes [10]. Although 88% of high school teachers and administrators reported being somewhat or very concerned about e-cigarette use by students, 34% of schools reported no formal communication from the school to parents about e-cigarette use [11].

Schools play an important role in initiation and prevention of nicotine and tobacco use among adolescents. Previous research suggests that higher rates of school smoking prevalence are associated with an increased risk of adolescent smoking [12, 13]. This influence may occur through a variety of mechanisms, including within school norms regarding tobacco, peer networks, socioeconomic characteristics, misconceptions about usage, and school-specific tobacco control policies [14–16]. However, schools also present an opportunity for intervention, as smoking-prevention programs can be administered in an environment where students are comfortable learning [17]. Simulation models have found that school-based interventions can reduce long-term nicotine dependency in a population [18].

Social network influences can have an effect on adolescent tobacco use. Friends within social networks can be leveraged to produce behavioral changes [19]. This is particularly true for youth, as they tend to emulate behavior of those whom they consider as a friend [20]. Further, network-based interventions can be provided in group settings, which help to reinforce adoption of behavioral change [19].

Using opinion leaders is an effective method of conducting school-based health programs. Intervention programs that are administered by central authority figures are met with more resistance when compared with opinion leaders that are peers [19]. Opinion leaders (also referred to as peer leaders or champions) are able to help modify social norms, which can change perceptions surrounding tobacco products [15]. These changes can be more resilient because the individual experiences changes in the behaviors that they perceive as normal [21].

Social network analysis (SNA) can effectively identify opinion leaders, where various methods can be used to identify, select, and train opinion leaders for health behavior interventions [22]. Identifying opinion leaders through SNA have the benefits of being informed by the full communication and social relational structure of the community as well as implementing a strategy that pairs leaders with those closest to them.

### Study objectives

This primary objective of our study was to assess the feasibility and acceptability of using SNA to identify student opinion leaders in schools and train them to conduct e-cigarette prevention programming for their peers.

## Methods

### Trial design

**Fig. 1:**
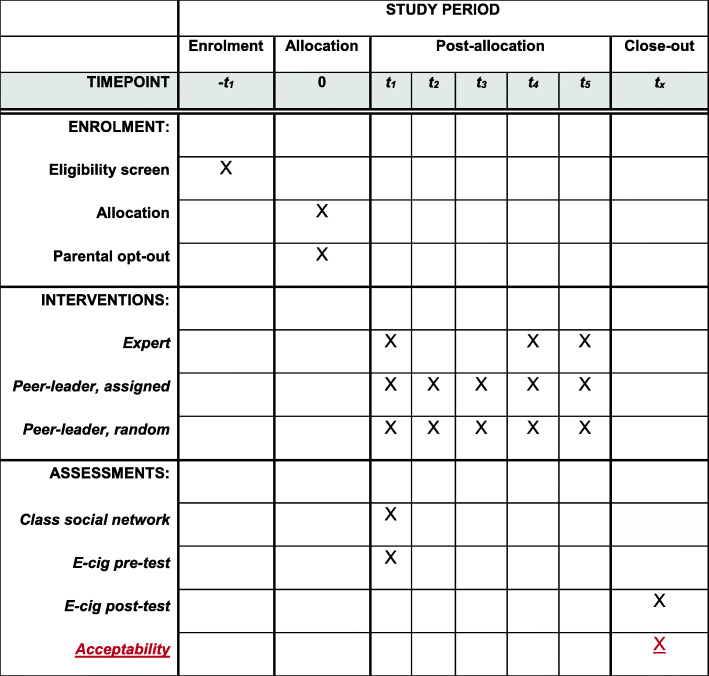
SPIRIT schedule of enrolment, interventions, and assessments

We conducted an open randomized controlled trial (RCT) with three arms—expert, elected peer-leader, and random peer-leader—for e-cigarette prevention programming. For additional information on this study’s compliance with Standard Protocol Items: Recommendations for Interventional Trials (SPIRIT), see Fig. 1 and Supplemental material.

### Participants and recruitment

We recruited nine schools in the Pittsburgh area comprised of 6th grade students (usually 11–12 years old) that are a heterogenous mix of gender, racial, and economic statuses (Table 1). Schools were conveniently selected based on referrals from colleagues that have existing relationships with administrators within each schools. No restrictions were placed on schools for other interventions being conducted. KHC and SM conducted informational meetings that typically included the principal, the guidance counselor, and the teacher of the class in which the intervention would occur. Following the meeting, the principal or classroom teacher would bring the proposed intervention to the school board. Upon approval from the school board, the research team worked with the schools to schedule days for the intervention at their convenience.

**Table 1:** School demographics

	Schools								
	S1	S2	S3	S4	S5	S6	S7	S8	S9
Female (%)	50	47	50	50	50	50	53	50	43
White (%)	90	66.4	32	63	84	93	93	88	84.7
Black (%)	4	22	65	32	6.8	< 1	2	2.8	8.5
Hispanic (%)	< 1	1.3	2	< 1	< 1	< 1	1	2.5	< 1
Asian (%)	1	< 1	< 1	< 1	< 1	< 1	< 1	1.7	< 1
Free lunch eligible (%)	45	57	100	98	50	49	39	29	30
6th grade students	126	174	266	98	231	93	92	194	299

### Randomization

An independent statistician generated an allocation table using Stata’s *ralloc* function, with schools stratified by demographic composition (delineated by greater or less than 67% white). That allocation table was provided to a research assistant, who assigned schools into the allocation table based on the order of date of agreement to participate in the study.

### Data collection and management

Consent documents were provided to students in paper form. A letter was sent to all 6th grade students’ parents/guardians describing the study. Parents/guardians were given a copy of each survey their child was asked to complete as well as an opt-out form should they choose not to have their child participate. Students provided verbal assent before the study began and were informed they could discontinue without penalty at any time. Students were not individually compensated for participation in the intervention; each participating class was provided with a party at the end of their participation. Additionally, each school that served as a study site was provided an honorarium.

All surveys were conducted by a member of the research team during regular school hours in a standard class period. Surveys were provided to students in paper form. The network survey and baseline e-cigarette survey were administered to students during the first session for all schools regardless of assignment. The final e-cigarette surveys were administered at the last day after the program had been completed (Fig. 1). Classroom teachers assisted the study team to ensure completion of surveys.

The principal investigator had the primary responsibility for study monitoring. Quarterly assessments were made of data quality and timeliness, participant risk/benefit ratio, protection of confidentiality of information, and any other factors that affected the study. The Institutional Review Board (IRB) would be informed immediately on a case by case basis, of any adverse outcomes, while requests for modifications of the protocol would be submitted to the IRB on a quarterly basis.

### Sample size

Given the nature of this study as a feasibility trial, sample size, and power calculations were not considered. The number of students were a convenience sample based on each of the nine schools (Table 1).

### Intervention overview

We based the e-cigarette prevention program on a combination of three existing programs: (1) UPMC Hillman Cancer Center Healthy Choices for Students School Program; (2) *CATCH My Breath*, an e-cigarette prevention for middle and high school students [23]; (3) Stanford’s *Tobacco Prevention Toolkit* [24]. This pilot study focused on modules that are most effective in reducing youth tobacco use, such as counter-marketing techniques, developing confidence, refusal skills, self-regulation, and normative education [23, 25–27].

Sixth grade students in each school completed a network survey that assessed the social networks in each class. Students listed the Roster ID numbers of up to five other students in four different network-based categories: closest friends, students that they think are the most popular, students that they view as the best leaders, and students they seek advice for personal concerns. Students also completed a pre-test e-cigarette survey. The items on the e-cigarette survey were adapted from the National Youth Tobacco Survey. For each school in peer-leader arm, we analyzed data from the social network survey to identify peer leaders in each classroom (schools in the expert-led arm were still given the social network survey). Once the peer-leaders were identified, we conducted 2 days of training to provide the skills necessary for them to lead other students in e-cigarette prevention programming. After completing the training, peer-leaders were assessed for their knowledge and understanding of the intervention.

During the 2-day intervention program, students in schools assigned to the elected peer-leader arm received the intervention from the trained student-nominated peer-leader (i.e., non-opinion leaders were matched with an opinion leader based on SNA). Students in schools assigned to the random peer-leader arm also received the intervention from the trained student-nominated opinion leader; however, the peer-leaders were randomly assigned. Students in schools assigned to the expert arm were administered the intervention by one of the study team members.

### Social network analysis

Within each class, we used degree centrality as the primary metric to identify peer leaders who are most likely to be influential change agents or promote attitudinal and behavioral change regarding e-cigarette use. Peer leaders were selected based on a weighted in-degree centrality ranking. Both the size of each group and the number of opinion leaders were informed by SNA and varied in each class; however, approximately 4–6 students were targeted to each peer-leader. Each of the nomination categories (i.e., friendship, opinion, academic support, and personal support) were evaluated to ensure the robustness of identification.

### Outcomes

Primary outcomes are satisfaction with the intervention program, including (1) appropriateness of the lessons, (2) peer-leader confidence, (3) students’ opinions of the lessons, and (4) student recruitment and retention rates. These data will be collected immediately after completion of the program. Secondary outcomes include intention-to-use and knowledge about e-cigarettes—based on items from the National Youth Tobacco Survey—will be collected before and after the intervention (Fig. 1).

### Data analysis

We will evaluate statistical properties of all data collected, including searching for outliers and missing data. As this study seeks to assess acceptability and feasibility, the primary data will include (1) recruitment rates, (2) retention rates, and (3) satisfaction with the intervention.

### Power calculations

As this study is a feasibility trial, it is inappropriate to consider mean outcomes due to lack of power. However, we will still calculate the standard deviation of the differences between the intervention and control groups.

## Discussion

This pilot trial is the first to evaluate the feasibility of leveraging social network analysis to identify 6th grade opinion leaders to lead a school-based e-cigarette intervention. The project was conducted in nine schools in the Pittsburgh area. We chose a pragmatic approach in order to inform implementation-related metrics for future studies to address the e-cigarette epidemic in US youth. If the pilot is successful and signals positive results, a larger fully-powered trial will be pursued to test the effectiveness of a peer-based approach.

The increase of teen e-cigarette use has the ability to create a new generation of nicotine-addicted persons. These nicotine-dependent youth have significantly greater odds of transitioning to traditional cigarettes compared to e-cigarette non-users, increasing their risk of cancer and cancer-related mortalities [19]. Previous research stresses the importance of preventing adolescent tobacco use as a method to prevent cancers and other chronic morbidities later in life. Using opinion leaders is an effective method of conducting school-based health programs. Preventing teen e-cigarette through innovative school-based programming can reduce the associated risks of developing tobacco-related cancer [17].

### Trial status

Protocol version: 4

Trial registration: ClinicalTrials.gov

Registration number: NCT04083469 (https://clinicaltrials.gov/ct2/show/NCT04083469).

Date of trial registration: September 10, 2019

Was this trial prospectively registered? Yes

Date recruitment began: August 2019

Completion date: July 2020

## Supplementary Information

**Additional file 1.**

## Data Availability

The datasets used and/or analyzed during the current study are available from the corresponding author on reasonable request.
